# A retrospective study of 72 cases diagnosed with idiopathic trigeminal neuralgia in indian populace

**DOI:** 10.4317/jced.51771

**Published:** 2015-02-01

**Authors:** Sunil Yadav, Hitesh-Chander Mittal, Akash Sachdeva, Ajay Verma, Vikas Dhupar, Anita Dhupar

**Affiliations:** 1 Professor and Head, Department of Dental Surgery, BPS Government Medical College for Women, Khanpur Kalan, Sonepat, Haryana, India; 2 Senior Resident, Department of Dental Surgery, BPS Government Medical College for Women, Khanpur Kalan, Sonepat, Haryana, India; 3 Reader, Department of Oral & Maxillofacial Surgery, Indraprastha Dental College, Ghaziabad, Uttar Pradesh, India; 4 Reader, Department of Oral & Maxillofacial Surgery, PDM Dental College & Hospital, Bahadurgarh, Haryana, India; 5 Professor & Head, Department of Oral & Maxillofacial Surgery, Goa Dental College & Hospital, Bambolim, Goa, India; 6 Professor, Department of Oral Pathology, Goa Dental College & Hospital, Bambolim, Goa, India

## Abstract

Context: Trigeminal neuralgia is as a chronic, debilitating condition, which can have a major impact on quality of life. There are few reports of trigeminal neuralgia in oriental populations. Objectives: To evaluate the retrospective data of the patients diagnosed with idiopathic trigeminal neuralgia and to understanding the disorder in the Indian populace. Methods: The retrospective data of 72 patients with typical idiopathic trigeminal neuralgia regarding age of onset, gender, site of involvement, clinical presentations and treatment received during three years of the follow up was collected and analyzed. Results: In the present retrospective study, the mean age was 54.9 years; female to male ratio was 2.13:1; rural to urban ratio 1.76:1 with 62.5% suffered trigeminal neuralgic pain on the right side. Carbamazepine was found to be highly effective in 60.8% of the cases on long-term basis with maintenance doses. Other treatment modalities were employed in more refractory cases including add-on of gabapentin, which relieved the symptoms for an additional duration of 13±3months. The neurolytic alcohol bloc was given in 30% of patients who stopped responding to combination of carbamazepine and gabapentin and relieved pain for a mean duration of 17.25±2.95 months. Twenty three percent of the patients (23%) required peripheral neurectomy. Conclusions: Carbamazepine was found to be highly effective in trigeminal neuralgia. Other treatment modality includes add-on of gabapentin, neurolytic alcohol blocs and peripheral surgical intervention in more refractory cases. Only limited cases needed further neurological consideration.

** Key words:**Trigeminal neuralgia, carbamazepine, gabapentin, alcohol bloc, peripheral neurectomy.

## Introduction

Trigeminal neuralgia is as a chronic, debilitating condition resulting in brief and intense episodes of facial pain in the distribution of one or more branches of the fifth cranial nerve (1-3). The episodes of facial pain are sporadic, sudden and often like “electric shocks”, lasting from a few seconds to several minutes. Etiology may be either idiopathic or secondary to intracranial lesions such as tumor, infarction and multiple sclerosis. Most of the cases are of idiopathic type with no underlying cause. Intracranial lesions that cause compression or traction of the trigeminal nerve are uncommon, but are a recognized cause of secondary trigeminal neuralgia. Patients with multiple sclerosis may develop trigeminal neuralgia; however, it is relatively rare. Trigeminal neuralgia is sometimes misdiagnosed due to non-availability of clear physical or laboratory diagnosis; and many a times, patient seek the help of numerous clinicians before a firm diagnosis is made. Though a benign disorder, it can have a major impact on quality of life and even gets refractory to various treatment modalities after some time.

Trigeminal neuralgia is rare and statistical data regarding it is limited. The estimated annual incidence of trigeminal neuralgia is 12.6 per 100000 persons per year (4) and its incidence increases with age. Although peak onset occurs between age 50 and 70 years, the disorder can also occur in children. Early literature suggested a strong preponderance in women; however, current data indicate that only approximately 60% of patients with trigeminal neuralgia are female (5). The annual incidence for women is approximately 5.9 cases per 100,000 women; for men, it is approximately 3.4 cases per 100,000 men (6).

Unfortunately, there is no definitive cure for trigeminal neuralgia at present. Relapses and reoccurrences may occur with significant morbidity; however, plethora of medical and surgical treatment options do exist to alleviate the patient’s symptoms.

There are few reports of trigeminal neuralgia in oriental populations (2,4,7). Because of the paucity of Indian data on incidence and management, a retrospective study of patients was undertaken with the purpose of understanding the disorder in the local context, and to compare with published data.

## Material and Methods

The present retrospective study relates to a consecutive series of 72 patients with typical idiopathic trigeminal neuralgia reported in the Department of Maxillofacial Surgery at the PDM Dental College Bahadurgarh, Haryana, India. Data for the study was retrieved from clinical patient records of patients reported during November 2009 to November 2010 and having been followed regularly for a minimum of three years. Only those records that had attached informed consent for the treatment and teaching/research purpose were included for retrieval of data. The inclusion criteria were based on mentioned clinical and radiological diagnosis of typical idiopathic trigeminal neuralgia; and a positive diagnostic local anesthetic test in the patient record sheet. The data regarding age of onset, gender, site of involvement, clinical presentations and treatment received during the follow up period was collected and analyzed.

## Results

The patient diagnosed with typical idiopathic trigeminal neuralgia in the present retrospective study ranged between 34 to 76 years, with a mean age of 54.9 years. The peak incidence was in the fifth and sixth decades of life ( Table 1). Females comprised 68 percent of the patients, representing a ratio of 2.13:1 ( Table 2). Majority of the patients belonged to the rural patients with a ratio of 1.76:1 as compared with urban population.

**Table 1:**
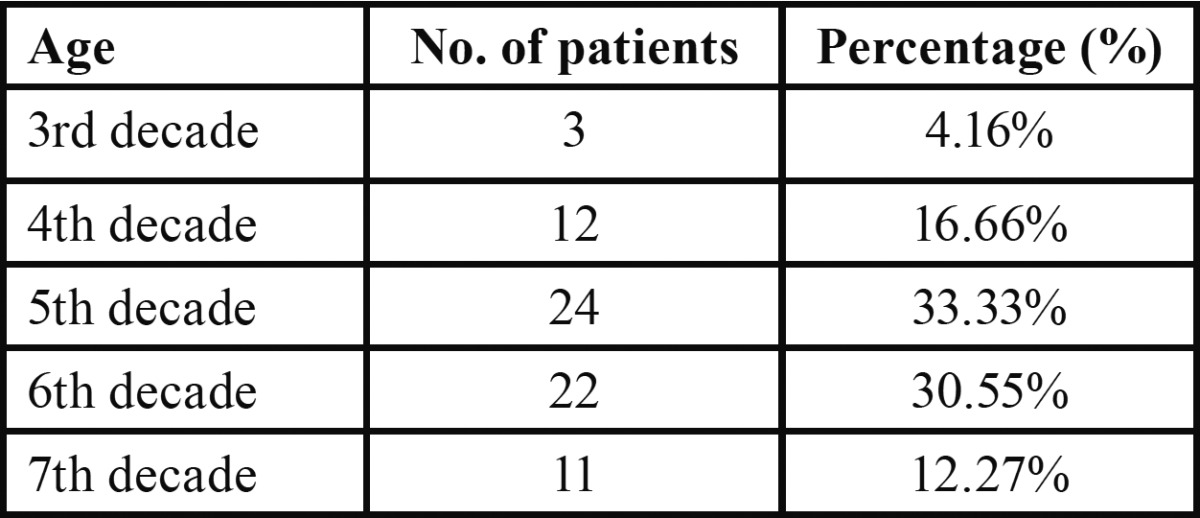
Age at onset of trigeminal neuralgia.

**Table 2:**
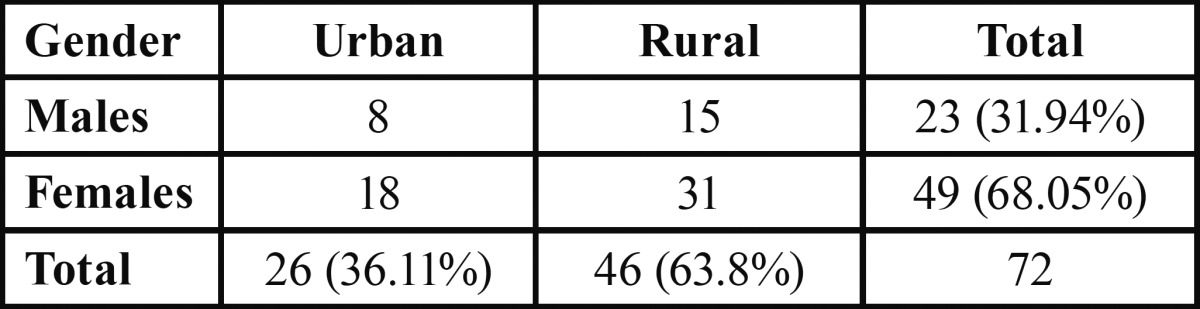
Distribution according to gender and community.

Of the 72 cases, 45 patients (62.5%) suffered excruciating pain on the right side of the face and 27 patients on the left side ( Table 3). This gave a site ratio of 1.6:1 confirming a predominance of right side facial affliction. The mandibular division was the most frequently involved branch ( Table 3). Forty-one patients (56.9 percent) reported neuralgic pain confined solely to the mandibular distribution of the face. Out of these reported mandibular division involvement cases, twenty-eight patients reported inferior alveolar nerve involvements while other thirteen-reported neuralgic pain only in the distribution of mental nerve. Twenty-seven patients (37.5 percent) reported neuralgic pain in the distribution of maxillary nerve, all with infra orbital nerve involvement. Four patients (5.6 percent) suffered from this condition with the involvement of the maxillary and mandibular division on the same side having involvement of mental and infra-orbital nerve involvement.

**Table 3:**
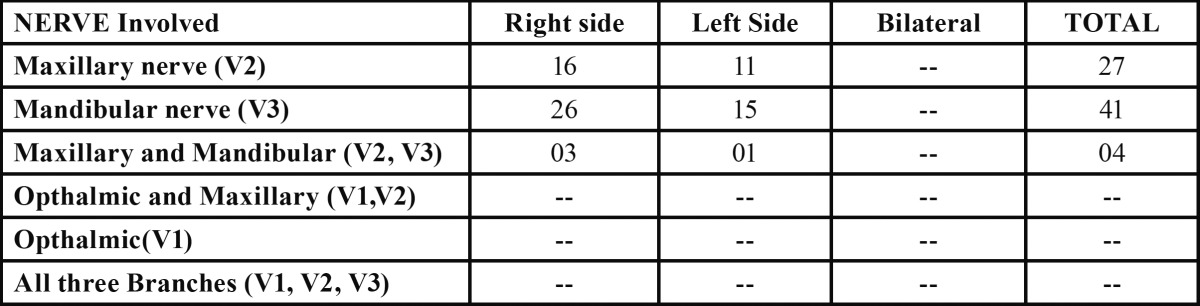
Distribution of side of face and division of nerve involved.

Treatment in 69 (95.8 percent) of the 72 patients ( Table 4) was started with carbamazepine and responded well symptomatically initially, and remaining 3 (4.2 percent) were directly treated with peripheral neurectomy because these cases were known to be refractory to other treatment modalities and were referred from other centers for surgical management. Over a period of four to six months, 27 (39 percent) of the patients given carbamazepine required supplementary medication i.e. gabapentin. After a mean period of 13.38±3.73 months, 30% of the patients receiving medicinal treatment became refractory and treated with absolute alcohol injection (0.5 to 0.8 ml) for the particular affected peripheral branch ( Table 4). Surgical modality (peripheral neurectomy) was employed to further ease the pain (23%) in 16 cases after a mean duration of 17.25±2.95 months. Out of 16 neurectomies, seven cases were operated for infra orbital, three cases for mental nerve and six cases for inferior alveolar nerve neurectomy respectively. However, 31% (n=5) of the patients underwent neurectomy required additional medicinal treatment after peripheral neurectomy in order to relieve symptoms. Out of these 25% (n=4) cases were referred to neurologists for further evaluation and management when developed symptoms within 6 months of surgical therapy.

**Table 4:**
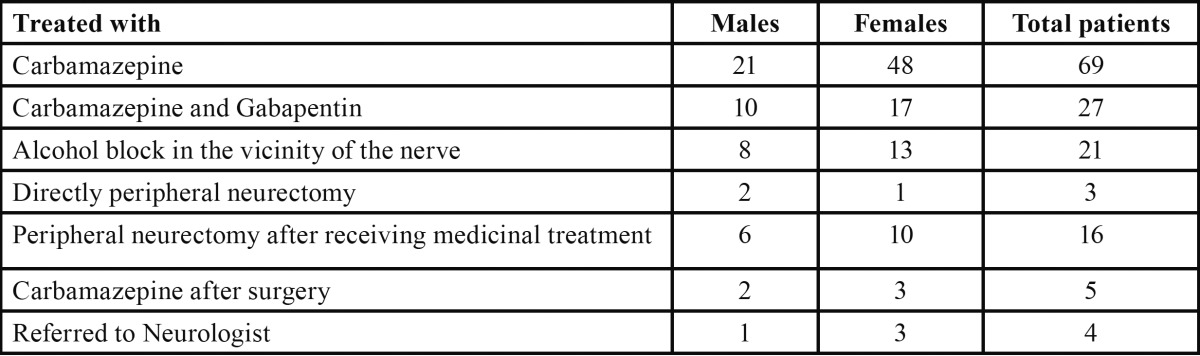
Treatment received by the patients with idiopathic trigeminal neuralgia.

## Discussion

Trigeminal neuralgia is an uncommon disorder seen in dental and neurologic practice, which presents with brief lancinating pain in the facial region in the area distributed by the trigeminal nerve. The disease is also known by less familiar names such as ‘Fotergill’s disease’ or ‘tic douloureux’. Trigeminal neuralgia can be classified based on etiology as primary or idiopathic and secondary or symptomatic.

The reported peak age of onset of trigeminal neuralgia is in fifth to eighth decades of life (6-8). Younger age has been found to be associated with symptomatic trigeminal neuralgia. However, considerable overlap in age ranges of patient with classical trigeminal neuralgia and symptomatic trigeminal neuralgia has also been reported (9,10). Similar trend was also observed in the present study with the peak age of onset between fifth and sixth decades of life.

In literature, female predominance has been reported in the ratio of 5.9:3.4 (6,8). Conversely, a male predominance has been reported in three reports from India (7). Although the disorder appeared to have a gender inclination, Zakrzewska (11) noted an equal representation of male to female incidence when adjustment was made to account for the older female population. However, the present study favors the trait of female dominance, with a ratio of 2.13:1 reflecting an elevated risk for female subjects.

In the present study, a rural predominance in the ratio of 1.76:1 compared to urban population was also observed. The one of possible reasons could be rural location of institute and thus easy accessibility to rural population. The other possibility could be hypothesized that rural females are more prone to domestic violence, poor nutrition ignored behavior and with time the accumulated psychological stress may have some role in etiology or precipitation of trigeminal neuralgia.

Most commonly it is unilateral, but 3% of cases with bilateral involvement have also been reported. Bilateral cases need special attention due to complexity of presentation and management (6,7,12). The trigeminal neuralgia usually affects right side of face as reported in literature (8,13,14) and, present study also justifies the same observation as right side of the face was affected in the ratio 1.6:1. Not even a single case of bilateral trigeminal neuralgia was found in the present study.

In the present series about two third (62.5%) of the patients found to be affected with involvement of mandibular division either alone or in combination with the maxillary division. The findings suggested mandibular division as most frequently affected branch of the fifth cranial nerve. The majority of published literature revealed that the mandibular division (V3) was most commonly involved and the ophthalmic division (V1) was less commonly presented (8,14-16). Shankland (15) reported that a third of the patients in their study presented with neuralgic pain involving both the second and third divisions of the fifth nerve. Approximately 6% of the patients in the present study also had involvement of both mandibular and maxillary divisions.

The possible reason could be related to the inherent higher prevalence with involvement of mandibular division in Asian population (8). The other possibility could be hospital bias yielded due to patients reporting to ophthalmologist for pain in the 1st division of 5th cranial nerve and does not report to maxillofacial or dental departments. Similarly, the patients with involvement of mandibular branch consults dental surgeon and those reporting to other specialty may be referred back to dental departments as the pain due to dental origin to be ruled out.

Treatment of trigeminal neuralgia ranges from simple medical to complex surgical management. The first line of defense is medicinal which includes carbamazepine and other related anticonvulsant drugs. These drugs work as Na+ channel blockers and relieve pain in trigeminal neuralgia by suppressing membrane resonance and firing in injured afferents.

Carbamazepine is the most studied and remains the drug of choice for treating trigeminal neuralgia (2,8,9,17-20). Treatment begins with 100 to 200 mg two to three times daily. Doses should be increased very progressively and titrated to the severity of the patient’s pain. In some cases a maintenance dosage of 200 to 400 mg per day is sufficient to keep the patient pain free. Studies have shown that it is effective in long-term treatment in 75% of the patients in whom the drug was initially effective (17). The controlled clinical trials showed to reduce pain severity, number of spontaneous paroxysms, and number of trigger (18). The pre-sent study also showed its effectiveness in 60.8% of the cases on long-term basis with maintenance doses.

In cases, where paroxysms of pain persist with therapeutic blood levels of carbamazepine, the second-line approach is add on therapy with baclofen, gabapentin, pregablin (8), lamotrigine, clonazepam, sodium valproate, phenytoin sodium, opiates (21). However, formal conclusive studies are still lacking for these combination therapies. In present study, combination of carbamazepine and gabapentin was given to 39% (n=27) patients who failed to respond with carbamazepine alone. Twenty-one patients (30%) stopped responding to combination of carbamazepine and gabapentin after an additional duration of 13±3months. Solaro *et al.* (22) in cases of trigeminal neuralgia with multiple sclerosis observed similar findings where co-administered carbamazepine and gabapentin found to deliver synergistic effect on trigeminal neuralgia. Prisco *et al.* (23) also evaluated combination therapy (carbamazepine + gabapentin or carbamazepine + pregabalin) in three patients refractory to antiepileptic medications and observed significant improvement during one year of follow up. Cheshire (24) also concluded in their retrospective study that gabapentin could be effective as first or second line treatment of trigeminal neuralgia, even in cases resistant to traditional treatment modalities. In their study, pain relief was obtained in 43 patients and sustained in two thirds during a mean follow-up time of 8 months (24). Therefore, it could be hypothesized that the co-administration of carbamazepine and gabapentin has a synergistic effect on trigeminal neuralgic pain.

The patients who do not respond to medicinal treatment, some form of surgery should be proposed. It is estimated that up to 50% of the patient will sooner or later be in this situation (17). The first surgical group falls under the peripheral procedures, which include neurolytic alcohol block, neurectomy, radiofrequency thermocoagulation of the peripheral branches, cryotherapy and peripheral acupuncture. The studies on the peripheral procedures showed that 50% of the patients had a recurrence of pain after one year but the morbidity is low (19). The complex surgical procedure includes rhizotomy, radiofrequency thermocoagulation and decompression of ganglion or main nerve trunk. There are no randomized controlled trials to guide comparisons between the surgical options available.

Alcohol injections are useful in those who are refractory to drug therapy and in whom surgery is delayed or defer for any reason. In a retrospective study, the distal injection technique relieved pain for a mean time interval of 14.13 ± 8.66 months. There was a fall in the duration of effect with subsequent injections (25). Another retrospective study was conducted from 1994-1999 by McLeod *et al.* (26) and found that peripheral alcohol injection lasted for a mean of 11 months. However, a failure rate of 10% has been reported (26). In the present study, alcohol injection relieved pain for a mean duration of 17.25±2.95 months. The combination of efficacy and reduced morbidity makes this procedure preferable for the treatment of trigeminal neuralgia.

In cases, where medicinal and alcohol block is ineffective or refractory with involvement of only one branch, peripheral neurectomy is a safe surgical method. The reported success rate of peripheral neurectomy in few controlled studies ranges from 26%-75% over different time intervals (2). In contrast to these studies, one study found recurrence in 78% of patients who had undergone neurectomy during a mean follow up of 7 years. One‐half of these patients had their first recurrence within a month (27). In the present study, 16 patients (23%) required peripheral neurectomy and most patients within a few days have a decrease in pain severity or no pain at all. The complication of concern is the sensory loss in the area. The patients who had underwent surgery; the majority of them have preferred to have surgery earlier, similar to reported studies by Zakrzewska *et al.* (28). However, five patients (31%) required additional medicinal treatment to control the pain. Out of these five patients, four were referred to neurosurgeon with in six months of peripheral neurectomy. Patients who have successful surgeries often need secondary or tertiary surgeries on different trigeminal nerve branches due to migration of pain.

Medical treatment of trigeminal neuralgia is usually the first option; and Carbamazepine was found to be highly effective and specific for this condition. Other treatment modalities were employed in more refractory cases including add-on of gabapentin and neurolytic alcohol blocs. Simple surgical intervention was used to relieve the intractable pain of those few patients who did not respond to medical therapy. Only a few cases needed further neurological consideration.
